# Clinical impact of 99mTc-HDP SPECT/CT imaging as standard workup for foot and ankle osteoarthritis

**DOI:** 10.1259/bjro.20230017

**Published:** 2023-08-29

**Authors:** AJ van Hasselt, J Pustjens, AD de Zwart, M Dal, AJ de Vries, TM van Raaij

**Affiliations:** 1 Department of Orthopaedic Surgery, Martini Ziekenhuis, Groningen, Netherlands; 2 Department of Orthopaedics Surgery, Universitair Medisch Centrum Groningen (UMCG), Groningen, Netherlands; 3 Department of Nuclear Medicine, Martini Ziekenhuis, Groningen, Netherlands

## Abstract

**Objective:**

The primary aim of this study was to assess to what extent 99mTc-HDP Single photon emission computed tomography/computed tomography (SPECT/CT) will lead to change of diagnosis and treatment, in patients with suspected foot and ankle osteoarthritis (OA). Secondary aim was to assess the intraobserver variability.

**Methods:**

Retrospectively 107 patients, with suspected foot and/or ankle OA of which a SPECT/CT was made, were included for analysis. All the clinical and radiological data were randomized and blinded before being scored by one experienced orthopaedic surgeon. Firstly, based on the clinical data and conventional radiographs, a diagnosis and treatment plan was scored. Secondly, the observer accessed the SPECT/CT and could change the diagnosis and treatment plan. Additionally, the intraobserver reliability was determined by data of 18 patients that were added in twofold to the dataset, without awareness of the observer and by calculating the κ values.

**Results:**

The diagnosis changed in 53% (57/107) and treatment plans changed in 26% (28/107) of the patients. Intraobserver reliability for the conventional workup was *k* = 0.54 (moderate strength of agreement), compared to *k* = 0.66 (substantial strength of agreement) when SPECT/CT data were added.

**Conclusions:**

This study describes the influence of SPECT/CT on diagnosis and treatment plans in patients with suspected symptomatic OA. Also, it shows SPECT/CT leads to a higher intraobserver variability. We believe SPECT/CT has a promising role in the workup for foot and ankle OA.

**Advances in knowledge:**

In addition to what was found in complex foot and ankle cases, this study shows that in patients with non-complex foot and ankle problems, SPECT/CT has a substantial influence on the diagnosis (and subsequent treatment plan).

## Introduction

Due to the complex anatomy of the foot and ankle, accurate localisation of symptomatic osteoarthritic lesions can be difficult, especially in the subtalar and midfoot regions of the foot.^1–4^ 99mTc-HDP Single photon emission computed tomography/computed tomography (SPECT/CT) is an upcoming diagnostic modality in orthopaedics and literature shows promising results for the use of detecting symptomatic osteoarthritic lesions.^5–9^ For the use of SPECT/CT in osteoarthritic lesions in the foot and ankle specifically, few articles have been published showing that the use of SPECT/CT leads to a change in diagnosis between 70 and 90%.^1,3,4^ In these studies, SPECT/CT’s were made for a variety of pathologies and concerned often complex cases. At our clinic, SPECT/CT is being used routinely in the workup for foot and ankle OA for all patients in which an operative treatment is considered. However, we questioned to what extend SPECT/CT influenced the diagnosis when used in this setting.

The primary aim of this study was to evaluate to what extent the use of SPECT/CT will lead to a different diagnosis and treatment plan compared to conventional workup (full history, physical examination and radiographs), in non-complex group of patients with suspected symptomatic foot and/or ankle OA. Secondary aim was to measure the intraobserver reliability for conventional workup alone and for conventional workup combined with SPECT/CT.

## Material and methods

### Patients

Eligible patients were those treated by a single orthopaedic surgeon specialised in foot and ankle surgery at a teaching hospital setting in the Netherlands between January 2013 and January 2018. The inclusion criteria for this study were suspected symptomatic foot and ankle OA, complete medical history with detailed physical examination, availability of standard conventional radiographs (conventional workup) and a SPECT/CT. Exclusion criteria were; sustained foot and/or ankle trauma within one year of presentation, history of neurological disease or foot and/or ankle surgery, age younger than 18 and isolated forefoot pathology. The local medical ethics committee approved the study protocol.

### Image acquisition

SPECT/CT was performed using a hybrid system (Symbia T, six slice, Siemens, Erlangen, Germany), which consists of a pair of low-energy, high resolution collimators and a dual-head γ camera with an integrated 6-slice CT scanner. SPECT images of the feet with 550 MBq of ^99m^Tc hydroxymethylene disphosphonate (99mTc-HDP) were taken 4 h after intravenous injection using the step and shoot method, with matrix size of 128 × 128, 180° rotation, 48 views and 20 s per frame. Subsequently, the CT component was applied with 130 kV and 30 mAs. The images were reconstructed with 1.25 mm slice thickness.

### Data assessment

Patient data, consisting of age, body mass index (BMI), sex, full patient history, detailed physical examination, conventional radiographic and SPECT/CT imaging including a radiological report, were retrospectively collected from the hospital’s medical records. Radiographic images were evaluated by hospital radiologists and SPECT/CT was evaluated by hospital nuclear specialist (MD). Each patient was assigned a randomised number and all information was anonymised. All data were subsequently uploaded to the research management software ‘my-research manager’. Next the data were presented to the original treating physician: an orthopaedic surgeon (TVR) with over 10 years of experience in foot and ankle surgery (observer). First, the observer made a diagnosis and treatment plan based on the conventional workup including reviewing the radiographic images and the hospital radiologist official report. The hospital radiologist official report consisted of a description of degenerative lesions/joints. Second, the case was re-assesed with access to the SPECT/CT and the official report from the nuclear specialist. Official nuclear specialists report consisted of uptake *vs* no uptake per joint, description of degenerative lesions/joints and a suggestion of diagnosis based on SPECT/CT. From these data, we evaluated the amount of changes for both diagnosis and treatment. When diagnosis or treatments matched before and after addition of SPECT/CT data, this was deemed to be ‘in agreement’, if they differed it was ‘change in diagnose/treatment’.

To assess intraobserver reliability, data of 18 patients was added in twofold in randomised order and scored twice by the blinded observer – who was unaware of the duplicates – for standard workup and for SPECT/CT combined with standard workup. Intraobserver reliability was determined by calculating the κ values for conventional workup and after addition of SPECT/CT.

### Statistical analysis

Descriptive statistics were used to present the data. Since age and BMI were ratio/interval level and normally distributed, means, standard deviations and ranges were used. Frequencies were used with categorical data. For the intraobserver analysis for both the conventional and the SPECT/CT diagnosis, Cohen’s κ was used, where the diagnostic options were categorised. According to Landis and Koch, kappa<0 is considered poor, 0.01–0.20 is slight, 0.21–0.40 is fair, 0.41–0.60 is moderate, 0.61–0.80 is substantial, and 0.81–1.00 is almost perfect.^10^ The statistical analysis was performed using IBM SPSS version 20.0.

## Results

107 patients met the inclusion and exclusion criteria. Of the included patients, 50 were female and 57 were male. The average age was 60.1 years SD 12.1 (range 18–79) and the average BMI 29.9 kg SD 4.8 (range 20.6–46.8).

### Change of diagnosis

The diagnosis was changed for 57/107 patients (53%) after additional evaluation of SPECT/CT data. The largest subgroup was diagnosed with talocrural OA: 26 patients (24%) after conventional workup and 32 patients (30%) with the data of the SPECT/CT included. The group of patients in which the diagnosis changed the most were those with tarsometatarsal OA (81% change of diagnosis) (Table 1). The least amount of change was in the patients diagnosed with talocrural- (31%) and subtalar (13%) OA.

**Table 1.** Frequencies of patients with diagnosis options after conventional workup (first two columns), and the diagnosis after the interpretation of the SPECT/CT. Differences in diagnosis between both workups were presented as percentages (percentage of changed diagnosis). The numbers in bold represent the patients in which the observer was in agreement for the two work up’s

Change in diagnosis																
Diagnosis based on conventional workup alone		Diagnosis conventional workup and SPECT/CT combined														
Joint	Patients (n)	NA	TC	TC + ST	TC + ST+TN	TC+other	ST	ST + TN	TN	TN+other	TMT	≠TMT	TMT+other	Other	≠Other	Percentage of changed diagnosis
NA	10	**3**	2											5		70%
*TC*	26	1	**18**	5	1		1									31%
TC + ST	18		8	**8**		1	1									56%
TC + ST+TN	5		2	1	**1**	1										80%
TC+other	6		1	1		**1**	1	1						1		83%
ST	8		1				**7**									13%
ST + TN	2							**1**	1							50%
TN	8						2		**3**	2	1					63%
TN+CC	1									**1**						0%
TMT	16	2									**3**	8	3			81%
Other	7								1		1			**4**	1	43%
Total	107	6	32	15	2	3	12	2	5	3	5	8	3	10	1	53%

CC, Calcaneocuboid; NA, No Abnormalities; ≠Other, Different other diagnosis Conventional workup: Full patient history, physical examination and conventional radiographs; SPECT/CT, SPECT/CT imaging including a radiological report, Bold printed values indicate no change of diagnosis; ST, Subtalar; TC, Talocrural; TMT, Tarsometatarsal; ≠TMT, Different TMT; TN, Talonavicular.

### Change of treatment

SPECT/CT resulted in a change of treatment in 28/107 patients (26%). Non-operative treatment was chosen for 55/107 (51%) after evaluating the conventional workup. After SPECT/CT an alternative, non-operative treatment was chosen for 14/55 patients (25%) and operative treatment was suggested for 6/55 patients (10%). For the remaining 35/55 patients (63%) in this group, the observer suggested the same treatment before and after evaluating the SPECT/CT.

After conventional workup alone, operative treatment was suggested for 52/107 patients (49%). After addition of the SPECT/CT, operative plans changed for 6/52 patients (12%) and for 2/52 patients (4%) it was changed to a non-operative treatment (Table 2).

**Table 2.** Frequencies of patients with a conservative (left side-of the table) or operative (right side) treatment after interpretation of the conventional workup (bold). Differences in treatment between both workups were presented as percentages (percentage of changed treatment plans)

Change in treatment										
		Conventional workup alone	Conventional workup and SPECT/CT combined			Conventional workup alone	Conventional workup and SPECT/CT combined			
Joint	Patients (n)	Conservative treatment	No change in treatment	Different Conservative^*a*^	Surgical treatment	Surgical	No change in treatment	Different Surgical	Conservative treatment	Percentage of changed treatment plans
**NA**	10	**10**	5	4	1	-	-	-	-	**50%**
**TC**	26	**10**	7	2	1	**16**	15	1	*-*	**15%**
**TC + ST**	18	**6**	3	1	2	**12**	9	3	-	**33%**
**TC + ST+TN**	5	**2**	2	-	-	**3**	2	-	1	**20%**
**TC+Other**	6	**4**	3	1	-	**2**	1	1		**33%**
**ST**	8	**2**	1	-	1	**6**	5	-	1	**25%**
**ST + TN**	2	**1**	1	-	-	**1**	1	-	-	**0%**
**TN**	8	**3**	2	1	-	**5**	5	-	-	**13%**
**TN+CC**	1	-	-	-	-	**1**	1	-	-	**0%**
**TMT**	16	**13**	9	4	-	**3**	3	-	-	**25%**
**Other**	7	**4**	2	1	1	**3**	2	1	-	**43%**
**Total (n**)	107	**55**	35	14	6	**52**	44	6	2	**26%**

CC, Calcaneocuboid; NA, No Abnormalities; SPECT/CT, SPECT/CT imaging including a radiological report; ST, Subtalar; TC, Talocrural; TN, Talonavicular.

a Injection in different joint included. Conventional workup: Full patient history, physical examination and conventional radiographs

### Intraobserver reliability

For intraobserver reliability, 18 patients were scored twice by the observer. Intraobserver reliability for the conventional workup was *k* = 0.54 (95% CI 0.28–0.79) compared to *k* = 0.66 (95% CI 0.42–0.90) when SPECT/CT data were added. This translates into ‘moderate strength of agreement’ for the conventional workup compared to ‘substantial strength of agreement’ after additional SPECT/CT.^9^

## Discussion

This study demonstrates that in non-complex group of patients with suspected foot and ankle OA, SPECT/CT results in a change of diagnosis in more than 50%. The choice of treatment was changed in 26% of the patients. When assessing intraobserver reliability, we found that the reliability was substantial when SPECT/CT data were added compared to moderate for conventional workup alone.

Our results are consistent with current literature in demonstrating that SPECT/CT has added value for investigating foot and ankle OA. The impact of SPECT/CT on diagnosis and treatment, however, was lower than that reported in other studies. Claassen et al retrospectively evaluated the added value of SPECT/CT and found a change of diagnosis for 68.6% of all patients.^1^ Kumar et al found an even higher impact on diagnoses of 75% in their retrospective study.^4^ This lower level of impact of SPECT/CT compared to other studies can be explained by the difference in patient selection. While Kumar et al and Claassen et al included only patients whose diagnosis was difficult with physical examination and plain radiographs alone, we have included all patients with suspected foot and ankle OA in which an operative treatment was considered. Concerning the different anatomical regions of the foot and ankle, Claassen et al found that the highest changes are diagnoses of Lisfranc (75%) and Chopart (75%) injuries and specifically suggest using SPECT/CT for lesions at the level of these joints. Our results support these findings since there was a high degree of change in diagnoses for the talonavicular (63%) and tarsometatarsal (81%) joints. When isolated talocrural or subtalar OA were suspected, SPECT/CT resulted in the least amount of change in diagnosis, at 31 and 13%, respectively. When lesions are suspected in these areas, SPECT/CT may be of lesser value.

Comparing our results of the observer variability with literature, high reliability of SPECT/CT was demonstrated by Pagenstert et al, who evaluated 20 patients with foot and ankle pain to assess intraobserver reliability of SPECT/CT as a single parameter compared with CT and bone scintigraphy.^2^ They concluded that SPECT/CT had excellent intraobserver variability (κ = 0.86; 95% CI 0.81–0.88), which was significantly higher than the intraobserver reliability for CT, bone scintigraphy, and CT plus bone scintigraphy. Claassen et al demonstrated that for SPECT/CT, when taking into account the clinical data – as was done in this current study ‒ the overall intraobserver and interobserver reliability is ‘good’ (*k* = 0.68), while for MRI it was found to be ‘fair’ (*k* = 0.38).^3^ Our results of the intraobserver analysis for the diagnosis using SPECT/CT (*k* = 0.66) are similar to the results found by Claassen et al in suggesting the SPECT/CT has good interobserver reliability when taking clinical data in consideration too.

**Figure 1.**
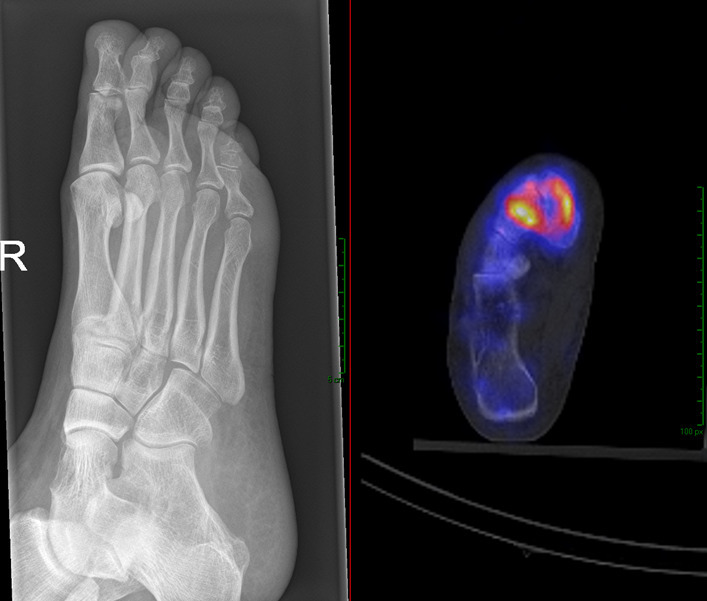
This patient (female of 50 years old) was treated 2 years before presenting for a stress fracture of the second metatarsal. However, her pain was persistent. On examination there was a mild swelling over the midfoot and there was tenderness when applying pressure at the midfoot. The X-rays did not show any abnormalities. The observer scored this case initially as no arthrosis. After assessing the SPECT/CT, the diagnosis was changed in navicular cuneiform OA and tarsometatarsal OA of the first ray. Before access to the SPECT/CT, the observer prescribed painkillers, after the SPECT/CT the plan was an ultrasound guided injection.

**Figure 2.**
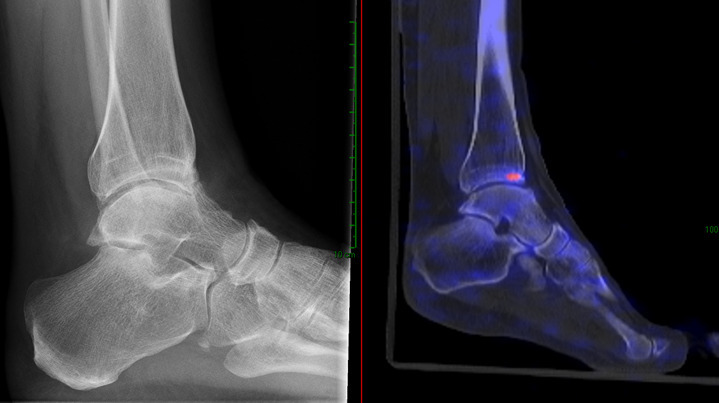
This patient (male of 64 years old) has a premedical history of excision of a chondral lesion and a corrective osteotomy for equines around 30 years ago. Since 5 years, there is a progressive pain on the lateral side-of the foot and midfoot. On examination, the foot is in slight varus but is correctable, The foot can dorsoflex no more than 5 degrees because of stiffness. There is pain when applying pressure over the talocrural joint, subtalar joint, and talonavicular joint. The X-ray showed mild OA of the talocrural joint and the talonavicular joint. The observer scored this case as talocrural, subtalar and talonavicular OA. After the SPECT/CT, the diagnosis changed in only talocrural OA. The suggested treatment plan was an orthopaedic shoe and did not changed after evaluating the SPECT/CT.

**Figure 3.**
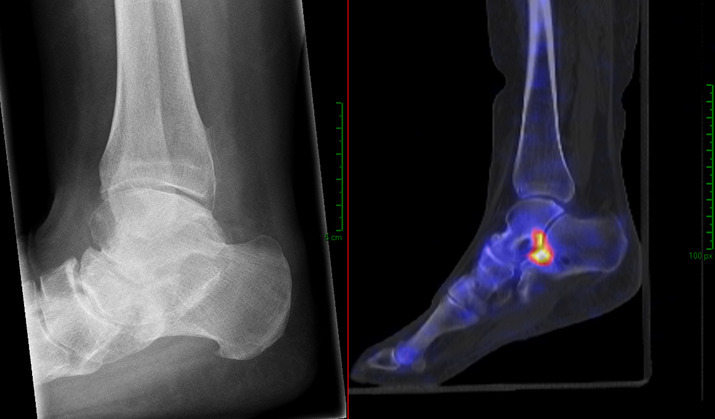
This patient (female of 60 years old) has pain of the foot and ankle for several years now. The ankle is stiff and painful, especially in the morning. On examination, the foot has valgus deformity and is not correctable. The talocrural joint has good function. Inversion is only 5 degrees and eversion goes up to 30 degrees. Not specifically painful. The X-ray showed OA of talocrural and talonavicular joint. The diagnosis initially was talocrural and talonavicular OA, while after the SPECT/CT it was subtalar OA. The treatment plan for this patient was an orthopaedic shoe and did not changed after the SPECT/CT.

**Figure 4.**
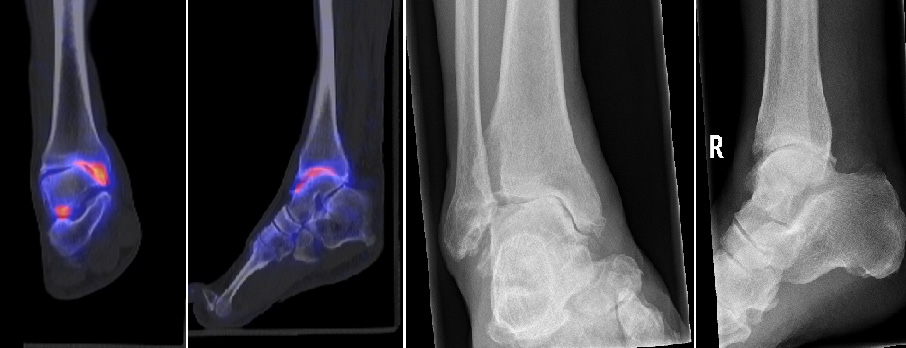
This patient is a 77-year-old male with no history of trauma. Since one and a half years, he has progressive pain and swelling of his right ankle after hiking and playing tennis. On examination, there is a mild varus axis and mildly swollen ankle. Although talocalcaneal is stiff, it is pain free on passive motion. There is mild pain while applying pressure subtalar on the medial side. The X-ray showed OA of the ankle with a asymmetrical joint line and possible subtalar OA. The diagnosis initially was talocrural and subtalar OA. SPECT/CT showed only OA and focal uptake in the sinus tarsi. The treatment plan changed from ultrasound-guided injection to an ankle arthrodesis.

Figures 1–4 show the potential of the SPECT/CT to exclude symptomatic OA, the potential to include symptomatic OA and to localise it more precisely. Although the results of the SPECT/CT look promising, the question remains if the changed diagnosis is the right one. However, there is no reference standard to define true symptomatic OA. An indication that the change of diagnosis by SPECT/CT may lead to a better outcome is provided by a study of Parthipun et al,^11^ who conducted a prospective study in which 52 patients with degenerative joint disease received joint injections, where the site of injection was determined using SPECT/CT findings. Their reference standard was a reduction of pain of more than 50%, which is commonly used in foot and ankle studies; 88% of patients demonstrated satisfactory pain relief according to the reference standard. Similar studies done with fluoroscopy^12^ and CT-guided^13^ injections that did not use SPECT/CT to determine the injection site showed a lesser reduction of pain of 64 and 57%, respectively. Kumar et al retrospectively included 50 patients where SPECT/CT was used to determine definitive diagnosis and treatment.^4^ Patients were treated non-operatively as well as operatively, and 92% displayed an improvement of symptoms.

Several limitations have to be considered for this study. The first and most important shortcoming is its selection bias, inherent to the retrospective design. We nonetheless believe our selection bias is limited compared to other studies because we used SPECT/CT relatively routinely. Moreover, as we excluded patients with a medical history of foot and ankle surgery or trauma, we left out the more complex cases and assessed a rather reproducible group of patients. Another limitation, which is also inherent to the retrospective design, is that an evaluation of the described clinical exam cannot be compared to a real life consult. However, the observer is a specialised foot and ankle surgeon and could interpreted his own relatively standardised notes reliably. As a result of the design of the study – single centre with only one observer – the interobserver reliability could not be determined. To our knowledge, there is no current literature about the interobserver reliability of the SPECT/CT and symptomatic foot and ankle OA. Finally, it is important to recognise that SPECT/CT will lead to increased radiation exposure. Total radiation exposure for SPECT/CT is approximated to be 5.5 mSv.^8^

## Conclusion

This study demonstrates that SPECT/CT significantly influences diagnosis and treatment when used routinely in foot and ankle OA. We believe we need prospective series with a well-defined reference standard to validate the diagnostic performance of SPECT/CT.
